# Successful Management of Pyoderma Gangrenosum in a Patient With Concurrent Behçet’s Disease and Familial Mediterranean Fever: A Case Report

**DOI:** 10.7759/cureus.68044

**Published:** 2024-08-28

**Authors:** Maroun Helou, Nael Al Irr

**Affiliations:** 1 General Practice, Faculty of Medicine and Health Science, Ramallah, PSE; 2 Internal Medicine, Palestine Medical Complex, Ramallah, PSE

**Keywords:** internal medicine, rheumatology, case report, familial mediterranean fever, behcet’s disease, pyoderma gangrenosum

## Abstract

Pyoderma gangrenosum (PG) is a rare ulcerative skin condition. Familial Mediterranean fever (FMF) and Behçet’s disease (BD) are autoinflammatory disorders common in Mediterranean populations. The coexistence of FMF and BD is unusual, and the presence of PG alongside both is even rarer.

We present a case of a 51-year-old male diagnosed with both BD and FMF who developed PG during treatment. His PG lesion was managed with a tailored regimen that included topical hydrocortisone acetate with fusidic acid, colchicine, topical tacrolimus, oral prednisone, and intravenous infliximab, which led to the successful healing of the PG lesion. This case underscores the need for specialized approaches to manage overlapping autoinflammatory conditions.

## Introduction

Pyoderma gangrenosum (PG) is a rare, ulcerative skin condition often associated with systemic diseases involving immune dysregulation [1]. Familial Mediterranean fever (FMF) is an inherited autoinflammatory disorder due to MEFV mutations; it is common in Mediterranean populations, marked by recurrent fevers and serositis [2]. Behçet’s disease (BD) is a multisystem inflammatory disorder also prevalent in these regions, characterized by recurrent oral and genital ulcers and uveitis. The coexistence of FMF and BD is uncommon, and the occurrence of PG with both conditions is even rarer [3].

We present a case of a 51-year-old male with a history of recurrent oral and genital ulcers, intermittent fever, joint pain, and uveitis who was diagnosed with BD based on clinical and laboratory findings. Concurrently, genetic testing revealed mutations in the MEFV gene, confirming the diagnosis of FMF. During treatment, the patient developed a rapidly progressing ulcer on his right calf, diagnosed as PG. His treatment regimen was adjusted to include topical hydrocortisone acetate with fusidic acid, colchicine, topical tacrolimus, oral prednisolone, and intravenous infliximab. The PG lesion healed completely within weeks.

## Case presentation

A 51-year-old male of Middle-Eastern descent presented with a history of recurrent, painful aphthous stomatitis and genital ulcers, which had been occurring for two years prior to presentation. The oral ulcers were initially treated with amoxicillin and a local anesthetic, which usually lasted for one week before healing, while the genital ulcers were managed with talc powder, persisting for two to three weeks before healing. The patient also reported intermittent fever, mainly at night, and multiple joint pains, especially at the knees, as well as painful swelling and enlargement in his left leg with a heaviness sensation that had started three months prior. Furthermore, he had a history of intermittent blurry vision in both eyes, for which he was diagnosed with uveitis by an ophthalmologist.

The patient had unilateral leg swelling, redness, dilated veins, and pitting edema, with the swelling extending up to the knee. Due to the suspicion of deep vein thrombosis (DVT), an ultrasound was performed. The ultrasound revealed dilated veins with decreased blood flow and compressibility in the superficial femoral vein (SFV) and popliteal vein, while the common femoral vein (CFV) showed normal blood flow, consistent with DVT. Consequently, the patient was started on subcutaneous Clexane (enoxaparin sodium) 1 mg/kg every 12 hours.

Rheumatologic laboratory investigations revealed positive lupus anticoagulants and HLA-B51 (tested by nested PCR technique from a DNA sample extracted from peripheral whole blood). The patient also had a positive anti-nuclear antibody ANA, which had a speckled pattern and a high titer of 1:640. Additional serological tests were negative, including rheumatoid factor (RF), PR3-ANCA, MPO-ANCA, anti-dsDNA, anti-SSA, and anti-Scl-70. Complement components C3, C4, and immunoglobulins were within normal ranges, and other tests revealed normal levels of immunoglobulins, normal T and B cell count, and normal complement function (CH50). The presentation of recurrent oral and genital ulcers, along with DVT and positive serological tests, led to a probable diagnosis of BD. Further investigations included a pathergy test, which was positive, fulfilling the criteria for the diagnosis of BD. The patient was started on a regimen of medications to control his symptoms, including azathioprine 50 mg daily and increasing the daily dose by 50 mg every four weeks as tolerated up to the target dose of 2.5 mg/kg/day, colchicine 1 mg/kg, prednisolone initial dose of 60 mg daily for four weeks, tapered over the ensuing six weeks, and anticoagulation.

A few months into his treatment regimen, the patient experienced recurrent, episodic, diffuse severe abdominal pain that had increased in frequency and severity and made him seek medical attention at our emergency department. The pain was accompanied by episodes of watery diarrhea without blood or mucus. His vitals were only remarkable for tachycardia (heart rate of 120 bpm); a physical exam showed a restless patient with severe abdominal tenderness mostly localized around the umbilicus. Laboratory investigations are demonstrated in Table 1. A complete blood count (CBC) showed only mild leukocytosis and a left shift of neutrophils, with an erythrocyte sedimentation rate (ESR) of 100 mm/h. To rule out any surgical causes for his pain, a CT scan was performed, and it showed no abnormalities. Colonoscopy and endoscopy were conducted, and they revealed only mild gastritis. Due to his recurrent severe abdominal pain and history of autoimmune disease, a diagnosis of FMF was highly suspected. Genetic testing for FMF was conducted and revealed heterozygous mutations in the pathogenic MEFV gene. He fulfilled the criteria used for diagnosis of FMF [2]. Thus, the patient had diagnoses of both FMF and BD.

**Table 1:** Laboratory findings Hgb, hemoglobin; MCV, mean cell volume; MCH, mean corpuscular hemoglobin; WBC, white blood cell; ESR, erythrocyte sedimentation rate

Laboratory parameter	Patient lab value	Reference range
Hgb	14	13.5-17 g/dL
MCV	86	80-100 fL
MCH	28	27-32 pg
WBC	14,000	4500-11,000/µL
ESR	100	<15 mm/h

**Figure 1:**
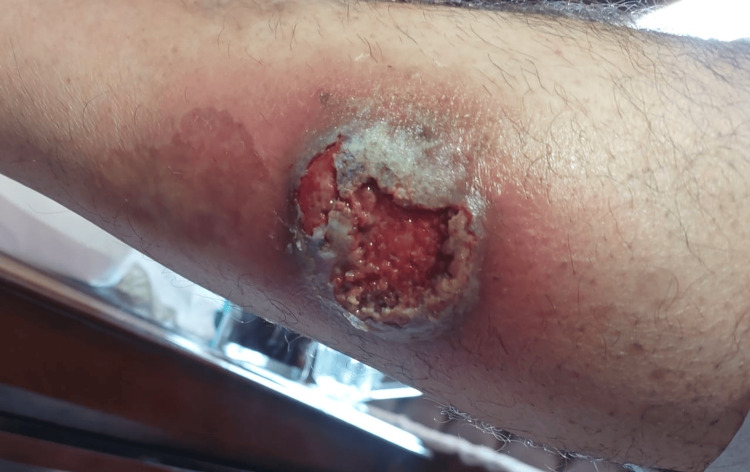
Pyoderma gangrenosum ulcer measuring 8 × 7 cm on the right leg

**Figure 2:**
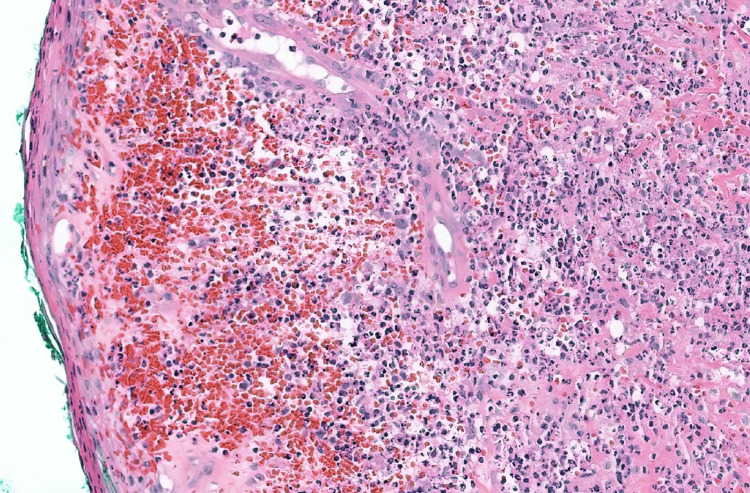
Dense neutrophil-predominant inflammatory infiltrate in the dermis with abscess formation

Two years into his treatment regimen, the patient noticed a nodule on his right calf that rapidly transformed into an ulcer measuring 8 × 7 cm (Figure 1). The ulcer was tender, with violaceous edges and undermined borders. The base was purulent and necrotic, extending to the subcutaneous fat. A biopsy revealed a dense neutrophil-predominant inflammatory infiltrate in the dermis with abscess formation (Figure 2), consistent with PG.

**Figure 3:**
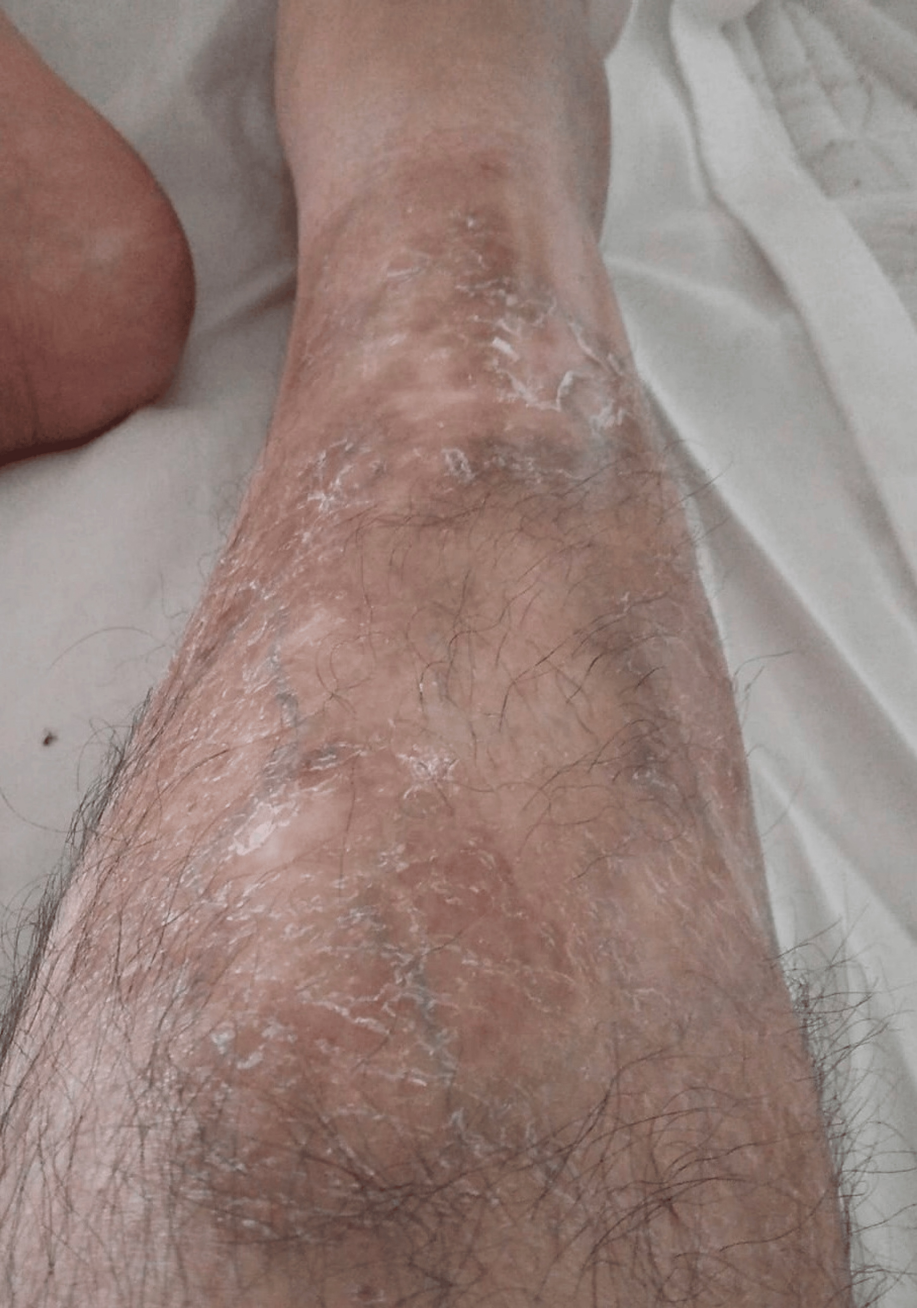
Healed pyoderma gangrenosum ulcer

His management plan was adjusted to include topical hydrocortisone acetate (10 mg) with fusidic acid (20 mg), topical tacrolimus (0.1% ointment) to be applied twice daily, and oral prednisone (1 mg/kg). Additionally, intravenous infusions of infliximab (5 mg/kg) were administered at 0, two, and six weeks, followed by maintenance infusions every six to eight weeks. Colchicine was continued to manage his FMF and BD. Remarkably, the ulcerative lesion that was diagnosed as PG began to heal within a few days and achieved complete healing within weeks (Figure 3).

## Discussion

FMF is primarily an autosomal recessive autoinflammatory disease, often presenting with recurrent bouts of fever and serositis, particularly in Mediterranean populations, including non-Ashkenazi Jews, Arabs, Armenians, and Turks [4]. This condition is associated with mutations in the MEFV gene, which encodes the pyrin protein. Pyrin is predominantly found in polymorphonuclear neutrophils and mature monocytes and regulates inflammation by enhancing interleukin (IL)-1 responsiveness. Mutations in the MEFV gene lead to increased pyrin inflammasome activity, resulting in more frequent inflammatory episodes. Typical symptoms include recurrent fevers, acute peritonitis, monoarticular synovitis, and, less commonly, pericarditis and amyloidosis. The most frequent mutations are p.Met694Val, p.Met694Ile, p.Val726Ala, p.Met680IleGC, and p.Glu148Gln [5]. These mutations exhibit geographical variability; for example, p.Met694Val and p.Met694Ile are the most prevalent mutations in Jordan and Lebanon [6].

BD is a chronic, systemic inflammatory disorder characterized by its relapsing and remitting nature, occurring in genetically predisposed individuals. First described in 1937 by Behçet, it is marked by recurrent oral ulcers, genital ulcers, and uveitis and is now recognized as a multisystem vasculopathy [7]. BD can affect various organs, including the musculoskeletal, cardiac, gastrointestinal, mucocutaneous, vascular, and urogenital systems. The disease typically manifests in the second and third decades of life and is more common in males. Similar to FMF, BD is prevalent in Middle Eastern and Mediterranean populations [3]. Like FMF, studies have shown a higher frequency of MEFV mutations in populations with BD, suggesting a potential genetic link between MEFV mutations and susceptibility to BD [5].

PG is a rare autoinflammatory ulcerative skin condition, with an incidence ranging from three to 10 cases per million individuals [8]. Due to the absence of a definitive diagnostic standard, the true prevalence of PG remains unclear, as it is often misdiagnosed. PG is classified as a neutrophilic dermatosis due to the significant neutrophilic inflammatory response observed in its lesions. The underlying mechanisms of PG are not fully understood and are believed to be multifactorial, involving both the innate and adaptive immune systems, as well as genetic factors. There is no universally accepted treatment protocol for PG, and effective management typically includes appropriate pain relief, wound care, and compression therapy. Treatment options may be topical or systemic, depending on various factors, such as the number, size, and location of lesions, the presence of comorbid conditions, potential side effects of medications, and patient preferences. It is important to note that non-compliance with therapy can result in severe complications. Ulcerative lesions may expand in size and depth, penetrating the subcutaneous fat and potentially exposing underlying tendons, ligaments, or even bones, which increases the risk of a life-threatening infection [9].

Clinically, PG manifests as ulcerated lesions, predominantly on the lower limbs. Patients typically present with one or more irregularly shaped ulcers featuring undermined edges and a distinctive gun-metal gray or purplish hue. These ulcers can vary in size and depth, often extending deeply enough to expose underlying muscles and tendons. There are also non-ulcerative variants, including pustular, bullous, and vegetative forms. Moreover, individuals with the M694V mutation are at an increased risk for developing inflammatory rheumatic diseases, such as chronic inflammatory seronegative spondyloarthropathy. A higher occurrence of chronic inflammatory seronegative arthropathy has been noted among patients with FMF. The coexistence of PG and FMF has been documented, and they are associated with mutations such as I1591T, M694V, and V726A in the MEFV gene. The primary pathophysiological mechanism linked to PG involves inflammasome activation and excessive production of IL-1, with occasional involvement of IL-17, a phenomenon also observed in FMF. This connection suggests that treatments targeting IL-1 and potentially IL-17 may be beneficial for both FMF and PG [1].

FMF and BD may coexist. The hallmark of autoinflammatory conditions is dysregulation of innate inflammatory mechanisms, resulting in vasculitic inflammation, recurrent fevers, and increased acute phase reactants. While FMF is a prototypical inherited autoinflammatory disease thought to result from inappropriate activation of the pro-inflammatory cytokine IL-1, the classification of BD into one of these categories remains unclear. Although BD has not been associated with autoantibodies, it is related to the HLA-B51 allele of class I MHC. Similar to FMF, there is increased activity of IL-1 and neutrophils in BD. Evidence suggests that BD is related to specific autoinflammatory diseases, particularly FMF. Notably, MEFV mutations have been shown to be prevalent in patients with BD.

To investigate the coexistence of FMF and BD, a literature review was performed, searching relevant articles using the terms “Familial Mediterranean fever and Behçet’s disease; FMF; Familial Mediterranean fever syndrome; Behçet’s disease” in the PubMed database. Nine case reports were identified, but evidence of a true association between FMF and BD is still lacking [5]. Schwartz et al. reported the coexistence of BD and FMF in 16 patients among 4000 FMF patients [10].

In the present case, lower extremity PG developed concurrently in a patient diagnosed with both BD and FMF. His management plan was adjusted and included a topical hydrocortisone acetate 10 mg with fusidic acid 20 mg, topical tacrolimus 0.1% ointment to be applied twice daily, and oral prednisone 1 mg/kg. Moreover, a 5 mg/kg intravenous infusion of infliximab was done at 0, two, and six weeks, followed by infusions every six to eight weeks. However, colchicine was continued as usual.

## Conclusions

This case highlights the rare coexistence of FMF, BD, and PG, underscoring the complexities in managing overlapping autoinflammatory conditions. The successful treatment emphasizes the importance of a comprehensive, individualized approach. Non-adherence to treatment can result in devastating outcomes, including death, making it crucial for both patients and clinicians to maintain strict adherence to management protocols. This rare occurrence necessitates heightened awareness and specialized management strategies, contributing to the growing body of literature and underscoring the need for ongoing research to optimize treatment protocols for these conditions.

## References

[1] Constantinou M, Parperis K (2022). Pyoderma gangrenosum in a patient with familial Mediterranean fever and chronic inflammatory seronegative arthropathy: a unique triad. BMJ Case Rep.

[2] Livneh A, Langevitz P, Zemer D (1997). Criteria for the diagnosis of familial Mediterranean fever. Arthritis Rheum.

[3] Mahaju S, Achhami E, Lamichhane S, Chalise KN, Gautam R (2023). A rare case of Behcet's disease in Nepal: multisystem manifestations and diagnostic challenges. Ann Med Surg (Lond).

[4] Watad A, Tiosano S, Yahav D, Comaneshter D, Shoenfeld Y, Cohen AD, Amital H (2017). Behçet's disease‬ and familial Mediterranean fever: two sides of the same coin or just an association? A cross-sectional study‬. Eur J Intern Med.

[5] Mir A, Ivory C, Cowan J (2023). Concurrence of familial Mediterranean fever and Behçet's disease: a case report and review of the literature. J Med Case Rep.

[6] Medlej-Hashim M, Serre JL, Corbani S (2005). Familial Mediterranean fever (FMF) in Lebanon and Jordan: a population genetics study and report of three novel mutations. Eur J Med Genet.

[7] Esatoglu SN, Kutlubay Z, Ucar D, Hatemi I, Uygunoglu U, Siva A, Hatemi G (2017). Behçet's syndrome: providing integrated care. J Multidiscip Healthc.

[8] McKenzie F, Arthur M, Ortega-Loayza AG (2018). Pyoderma gangrenosum: what do we know now?. Curr Derm Rep.

[9] Iliescu C, Popa L, Mihai M, Popescu MN, Beiu C (2024). Pyoderma gangrenosum: the impact of treatment non-adherence on disease progression. Cureus.

[10] Schwartz T, Langevitz P, Zemer D, Gazit E, Pras M, Livneh A (2000). Behçet's disease in Familial Mediterranean fever: characterization of the association between the two diseases. Semin Arthritis Rheum.

